# Rap Signaling in Normal Lymphocyte Development and Leukemia Genesis

**DOI:** 10.4110/in.2009.9.2.35

**Published:** 2009-04-30

**Authors:** Nagahiro Minato

**Affiliations:** Department of Immunology and Cell Biology, Graduate School of Medicine, Kyoto University, Sakyo-ku, Kyoto 606-8501, Japan.

**Keywords:** lymphocyte development, leukemia, RapGTPases, Spa-1, Notch

## Abstract

Although Rap GTPases of the Ras family remained enigmatic for years, extensive studies in this decade have revealed diverse functions of Rap signaling in the control of cell proliferation, differentiation, survival, adhesion, and movement. With the use of gene-engineered mice, we have uncovered essential roles of endogenous Rap signaling in normal lymphocyte development of both T- and B-lineage cells. Deregulation of Rap signaling, on the other hand, results in the development of characteristic leukemia in manners highly dependent on the contexts of cell lineages. These results highlight crucial roles of Rap signaling in the physiology and pathology of lymphocyte development.

## INTRODUCTION

Among the Ras superfamily G proteins, Rap GTPases (Rap1a, 1b, Rap2a, 2b, 2c) show the highest overall homology to classic Ras with an identical effector region (1,2). Rap GTPases are activated by a wide variety of external stimuli; the activation is mediated by specific guanine nucleotide exchange factors (GEFs), including C3G recruited by receptor protein tyrosine kinases, Epacs activated by cyclic AMP, and CalDAG GEFs activated by Ca^2+^ and/or diasylglycerol (3). On the other hand, the swift inactivation of RapGTP can be achieved with the aid of specific GTPase-activating proteins (GAPs), including Spa-1 family (Spa-1, Spa-L1, 2, 3) and RapGA1 (I, II) (3). In most cell types, Rap and Ras are activated concomitantly by many stimuli; however, they are under the distinct regulatory mechanisms, and Rap signaling may induce biological effects quite different from Ras signaling (4,5). In lymphocytes, Rap signaling is activated by various stimuli, including specific antigens, lymphokines, and chemokines. One of the most appreciated biological effects of Rap signaling is the regulation of cell adhesion; this effect is in part mediated by the activation of integrins (2,6,7). Through this effect, Rap signaling induces enhanced cell-matrix and cell-cell adhesion as well as cell migration (8). The effect is mediated partly by a Rap-specific effector called RapL, which associates with the intracellular domain of integrin α-units, mediating the inside-out activation of integrins on lymphocyte stimulation (9). Recent studies, however, indicate that the role of Rap signaling in lymphocytes goes beyond the regulation of cell adhesion and migration, affecting the cell fate decision in many cell types in the immune system. In this brief review, I shall summarize our recent studies on the crucial roles of Rap signaling in normal lymphocyte development as well as the disorders.

## ESSENTIAL ROLE OF RAP SIGNALING IN NORMAL LYMPHOCYTE DEVELOPMENT

Rap family consists of several highly related members with substantial functional redundancy, and this has been a major complication of the functional studies of Rap signaling. Recently, Rap1a-/- and Rap1b-/- mice were generated; however, they revealed surprisingly mild phenotypes (10,11). The results present a stark contrast with early embryonic lethality of C3G-/- as well as Spa-1 transgenic (Tg) mice, underscoring the functional redundancy among the Rap family members. To overcome the problem, we have investigated the role of Rap signaling in normal lymphocyte development with the use of conditional Tg mice for Spa-1 or dominant-negative Rap mutant (Rap1A17) capable of inhibiting the activation of all Rap family members in a physiological setting (12).

### T-lineage cell development

**Figure 1:**
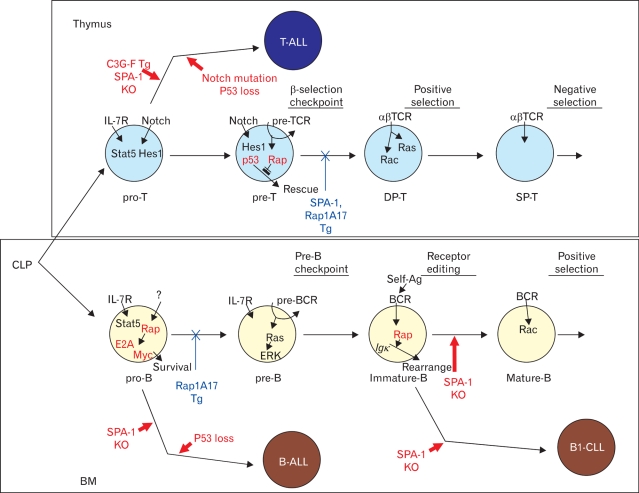
Rap signaling plays essential roles in normal lymphocyte development and leukemia genesis. Endogenous Rap signaling in T- and B-lineage cells has been conditionally modified by genetic manipulations; it is inhibited by the transgenic expression of SPA-1 or Rap1A17 (blue), and augmented by SPA-1 KO or C3G-F expression (red). Rap activation downstream of pre- TCR signaling is crucial to rescue the thymic pre-T cells with proper expression of TCRβ chains from p53-dependent cell death, called β-selection checkpoint. On the contrary, constitutive Rap activation in T-lineage progenitors results in the Notch-mutations and Notch-dependent T-ALL genesis. Rap signaling is also essential for the competence of IL-7-mediated survival and proliferation of pre-B cells in the BM. In addition, Rap signaling functions as a "self-sensing signal" in the immature B cells of BM to initiate B cell receptor (BCR) editing. The deregulated activation of Rap results in the acute B-CLL in collaboration with the loss-of function of p53 or B1-CLL associated with autoantibodies.

Conditional expression of Spa-1 in T-lineage cells (lck-Spa-1 Tg mice) resulted in a profound defect of thymic αβ T cell development without affecting γδ T cells (13). The thymic CD4/CD8 double-positive (DP) T cells were markedly diminished due to the arrested transition from double-negative (DN) stage 3 (CD25^+^ CD44^-^) to DN stage 4 (CD25^-^ CD44^-^), corresponding to β-selection checkpoint. In β-selection checkpoint, the thymocytes that have successfully rearranged TCRβ genes and expressed pre-TCR are rescued from cell death followed by the robust expansion via Notch-signaling, whereas those that have failed to do so are doomed to die via p53-dependent pathway (14,15). DN stage 4 thymocytes in the lck-Spa-1 Tg mice revealed a markedly enhanced apoptosis in situ, suggesting that Rap signaling was crucial for the rescue of pre-T cells from cell death. Indeed, it was confirmed that the ligand-independent, autonomous pre-TCR signaling activated both Rap1 and Rap2 in a DN thymocyte cell line. Furthermore, forced expression of farnesylated, constitutive active C3G (C3G-F) in Rag2-/- thymocytes induced their significant expansion in the presence of Notch-ligand (Delta-like 1)-expressing stroma cells, bypassing pre-TCR. And finally, it was found that lck-Spa-1 Tg mice at the p53-/- genetic background showed completely normal development of αβ T cells; the effect of p53 was gene dosage-dependent in that lck-Spa-1 p53+/- mice revealed a partial restoration of DP thymocyte development. These results unambiguously indicate that Rap signaling is essential in the rescue of pre-T cells from p53-mediated cell death in β-selection checkpoint. Currently, it remains unknown how the p53 checkpoint pathway usually associated with DNA damages is activated during pre-T cell development, and it also remains to be investigated how Rap signaling downstream of pre-TCR interferes with the p53-mediated cell death. We have confirmed that essentially identical phenotypes are found in lck-Rap1A17 Tg mice (Wakae, K and Minato, N, unpublished observation). Importantly, CD4-Spa-1 Tg mice showed normal αβ T cell development, indicating that Rap signaling is dispensable for αβ TCR-mediated positive selection (Fig. 1).

### B-lineage cell development

We also investigated the effects of Rap signaling in B cell development with the use of mb1-Rap1A17 Tg mice, which conditionally expressed a dominant-negative Rap1A17 mutant in B-lineage cells. The mb.1-Rap1A17 mice showed a marked diminution of BM pre-B cells, which exhibited impaired survival and expansion in response to IL-7 despite normal IL-7Rα expression (Katayama, Y et al., submitted for publication). The IL-7 responsiveness could not be restored by the expression of either constitutive active (CA) *Stat5a* or *CAPI3K-p100*; thus, the defect could be not attributable to the proximal signaling downstream of IL-7R. The results are consistent with the finding that lck-Spa-1 and lck-Rap1A17 Tg mice show completely normal γδT cell development, which is absolutely dependent on IL-7 (13). It is shown that an appropriate level of E2A, a bHLH transcription factor, is crucial for the competence of IL-7 response in pre-B cells (16). Thus E2A+/- mice reveal a significantly diminished development of pre-B cells, while E2A-/- mice show a complete defect in B-cell development (17). This effect is in part mediated by myc (c-myc and N-myc) proteins (16), which provide an essential driving force for the IL-7-mediated survival and expansion of B-lineage progenitors (BLP). We found that the mb.1-Rap1A17 mice showed a significantly reduced expression of E2A gene, and the impaired IL-7 responsiveness was restored by the transduction of c-myc gene. The results strongly suggest that Rap signaling plays an important role in sustaining the appropriate level of E2A in pre-B cells sufficient for the competence of IL-7 response. Accordingly, the mb.1-Rap1A17 mice showed markedly decreased mainstream B cells in the peripheral tissues with reduced numbers of follicles. However, the residual follicular B cells proliferated normally in response to the BCR stimulation and showed an efficient germinal center reaction following antigen immunization. Thus, Rap signaling plays a crucial role in pre-B cell development as well, whereas it is largely dispensable for the functioning of mature B cells *in vivo* (Fig. 1).

## DEREGULATED RAP SIGNALING AND LEUKEMIA

While the potential oncogenic effect of Rap signaling was a matter of arguments, the effect has been first revealed in Spa-1-targeted mice *in vivo*. Thus, Spa-1-/- mice developed a wide spectrum of leukemia with long latent periods, including myeloid, B-lineage and infrequently T-lineage leukemia (18). Because Spa-1is a principal Rap GAP in hematopoietic progenitors (HPC) (19), such leukemia genesis is attributed to the constitutive activation of endogenous Rap signaling in HPC. Given the crucial roles of Rap signaling in normal development of both T- and B-lineage cells, we addressed the involvement of deregulated Rap signaling in leukemia genesis.

### Notch-dependent T-cell acute lymphoblastic leukemia (T- ALL)

T cell leukemia was observed only infrequently in Spa-1-/- mice; however, it might well be due to the expression of other Spa-1 family genes (*Spa-Ls*) in the thymocytes compensating Spa-1. We therefore investigated the effects of deregulated endogenous Rap activation alternatively by the forced expression of C3G-F. Transplantation of the HPC retrovirally transduced with C3G-F into lethally irradiated mice resulted in marked thymic hyperplasia, consisting of blastic DP cells. These thymocytes were of oligoclonal origin, being reminiscent of multicentric thymoma (20). The thymic hyperplasia was more striking when Spa-1-/- HPC transduced with C3G-F were transplanted: around half of such recipients developed lethal systemic T cell leukemia invading most vital organs within 3 months (20). These leukemic cells were highly blastic DP cells of monoclonal origin, being compatible with the diagnosis of T cell acute lymphoblastic leukemia (T-ALL). The difference in the disease phenotypes, thymoma vs. T-ALL, paralleled with the extents of endogenous Rap activation; thus C3G-F^+^ Spa-1-/- HPC-derived T-ALL cells showed much higher RapGTP levels than C3G-F^+^ B6 HPC-derived thymoma cells. Interestingly, T-ALL cells so generated from C3G-F^+^ Spa-1-/- HPC all exhibited very high levels of an active form of Notch (1 and 3) along with the enhanced expression of Notch target genes such as Hes1, pTα, c-myc, and cyclin D1. Furthermore, their continuous proliferation *in vitro* was completely inhibited by a γ-secretase inhibitor, indicating the dependence of the leukemic growth on Notch signaling (20). The generation of Notch-dependent T-ALL was independent of pre-TCR, in agreement with the view that excessive Rap signaling synergized with Notch, bypassing pre-TCR signal (21,22). Accumulating evidence reveals a crucial role of Notch in human T-ALL. Although previously discovered Tan1, an active Notch IC via chromosomal translocation, is a rather rare event, recent studies indicate that the majority of human T-ALL show characteristic mutations in Notch gene at the two hot spots: HD (heterodimerization domain) and the PEST region containing a protein degradation target site (22). Also in murine models, T-ALLs developed in *Scl*-Tg and Ikaros-/- mice revealed Notch mutations at very high frequencies (23-25). Indeed, we have found that all the T-ALL cells developed from C3G-F^+^ Spa-1-/- HPC show insertional mutations at the PEST region of Notch gene, leading to the frame shift and premature termination (20). Importantly, all the mutations result in the loss of Thr residue at position 2512, a crucial phospholylation site for the recognition of an E3 ligase FBW7 affecting Notch (26). The reason why Notch gene mutations are induced in the thymocytes with excessive Rap activation remains to be investigated, and it also remains unknown whether the mutations are restricted to Notch gene or not. Nonetheless, these results provide the first indication that the deregulated small G protein signaling can be involved in T-ALL genesis (Fig. 1).

### B-cell leukemia and autoimmunity

Although the majority of leukemia in Spa-1-/- mice was of myeloid origin, a significant proportion (around 15%) of them developed chronic B-lineage cell leukemia. Interestingly, the leukemic cells were non-blastic lymphoid cells exhibiting CD5 and Mac1 expression, apparently corresponding to B1 cells (27). Indeed, the majority of Spa-1-/- mice showed a progressive increase in the peritoneal B1 cell population preceding overt leukemia development; this was associated with the generation of anti-dsDNA antibody and lupus-like glomerulonephritis (27). It has been shown that the excessive Rap activation downstream of BCR signaling on immature BM B cells enhances the secondary Igκ gene rearrangement and expression via p38MAPK/Creb-mediated *Oca*B induction, that is, BCR-receptor editing. Given that the receptor editing in the potentially autoreactive immature B cells is crucial to abrogate the autoreactivity, it is suggested that Rap signaling may function as a "self-sensing" signal that initiates the receptor editing upon encounter with self antigens. It is suggested that deregulated Rap signaling in such B cells of Spa-1-/- mice results in the excessive receptor editing, causing the accumulation of B1 cells with aberrant receptor editing and even Igκ/λ isotypic inclusion retaining the autoreactivity. In agreement, many of the *Spa-1-/-* mice with B1 cell-type leukemia also showed a hemolytic autoantibody, reminiscent of human B cell chronic lymphocytic leukemia (CLL) (Fig. 1). Interestingly, E2A+/- mice show the defective secondary IgL gene rearrangement and expression leading to the impaired receptor editing (17), thus it may be possible that aberrant E2A expression may also contribute to the excessive receptor editing in Spa-1-/- immature B cells. Most recently, we have found that Spa-1-/- mice are hypersensitive to X-ray radiation, and that their HPC reveal an enhanced p53 expression, implying the continuous genostresses (H. Tanaka and N. Minato, manuscript in preparation). In agreement with the findings, Spa-1-/- p53-/- mice rapidly develop acute leukemia at much shorter latent periods than Spa-1-/- mice, including highly aggressive T-lineage and B-lineage acute lymphoblastic leukemia (T, B-ALL) with gross chromosomal anomalies. (H. Tanaka and N. Minato, manuscript in preparation). Thus, excessive Rap signaling can highly predispose the malignant transformation of lymphoid cells, rapidly inducing acute blastic leukemia when combined with the loss-of-function of tumor suppressor genes (Fig. 1).

## CONCLUSION

Because of their structural similarity to Ras, the possible involvement of Rap in oncogenesis has been investigated extensively (28). Although it is unlikely that Rap functions as a classic oncogene that bypasses the various growth factors like classical Ras, a series of our genetic studies revealed crucial roles of Rap signaling in a wide variety of leukemia. Our recent studies, on the other hand, indicate that Rap signaling is crucially involved in normal development of both T- and B-lineage cells. Therefore, it seems quite likely that the deregulated Rap signaling contributes to the leukemogenesis as direct consequences of the disturbed lymphocyte development. The molecular mechanisms of leukemia genesis may vary depending on the contexts of cell-lineages and developmental stages, including the interaction with cell type-specific factors. Our recent results further suggest the involvement of excessive Rap signaling in the genetic instability of lymphocytes. In human leukemia, the involvement of classical Ras mutations is a relatively rare event, and thus further analysis on Rap signaling in leukemia may provide a new insight into the leukemia genesis in humans.
